# Reconstructing ancestral gene orders with duplications guided by synteny level genome reconstruction

**DOI:** 10.1186/s12859-016-1262-8

**Published:** 2016-11-11

**Authors:** Ashok Rajaraman, Jian Ma

**Affiliations:** Computational Biology Department, School of Computer Science, Carnegie Mellon University, 5000 Forbes Avenue, Pittsburgh, 15213 USA

**Keywords:** Ancestral genome reconstruction, Gene orders, Synteny blocks, Duplications

## Abstract

**Background:**

Reconstructing ancestral gene orders in the presence of duplications is important for a better understanding of genome evolution. Current methods for ancestral reconstruction are limited by either computational constraints or the availability of reliable gene trees, and often ignore duplications altogether. Recently, methods that consider duplications in ancestral reconstructions have been developed, but the quality of reconstruction, counted as the number of contiguous ancestral regions found, decreases rapidly with the number of duplicated genes, complicating the application of such approaches to mammalian genomes. However, such high fragmentation is not encountered when reconstructing mammalian genomes at the synteny-block level, although the relative positions of genes in such reconstruction cannot be recovered.

**Results:**

We propose a new heuristic method, MultiRes, to reconstruct ancestral gene orders with duplications guided by homologous synteny blocks for a set of related descendant genomes. The method uses a synteny-level reconstruction to break the gene-order problem into several subproblems, which are then combined in order to disambiguate duplicated genes. We applied this method to both simulated and real data. Our results showed that MultiRes outperforms other methods in terms of gene content, gene adjacency, and common interval recovery.

**Conclusions:**

This work demonstrates that the inclusion of synteny-level information can help us obtain better gene-level reconstructions. Our algorithm provides a basic toolbox for reconstructing ancestral gene orders with duplications. The source code of MultiRes is available on https://github.com/ma-compbio/MultiRes.

**Electronic supplementary material:**

The online version of this article (doi:10.1186/s12859-016-1262-8) contains supplementary material, which is available to authorized users.

## Background

Recent advances in next-generation sequencing technologies have dramatically expanded the reach of genetic studies to many more non-model organisms. Ancestral genome reconstruction based on the whole genome sequences of these new genomes will provide us with great opportunities to elucidate the trajectory of genome evolution and shed new light on the molecular signatures of phenotypic variation [1, 2]. The problem of predicting ancestral genome structures, in terms of ancestral gene orders [3] and synteny orders [4], has received much interest in comparative genomics [5–11]. Current methods for reconstructing ancestral gene orders often rely on the gene orders in extant species and their phylogeny to find a solution to optimize a relevant objective function. These methods are generally classified as (i) model-based approaches, which minimize genomic distances along all branches of a phylogeny [5, 12–14], where the distances are based on rearrangement events, such as inversion, indels, transposition and translocation; and (ii) model-free approaches, which maximize conserved syntenic characters in the descendant species [15, 16].

However, these methods usually do not account for insertions, duplications and losses [15, 17, 18]. While progress has been made to incorporate insertions and deletions [19], efficient reconstruction of gene orders with duplications remains a largely open problem. There exist maximum likelihood methods that also reconstruct gene orders with duplications [20], as well as methods which utilize reconciled gene trees into the reconstruction framework [21], but obtaining robust gene trees itself is a complicated problem [22]. A number of studies also show that incorporating duplications in current reconstruction models renders the related optimization problems computationally intractable [23, 24].

Reconstructing the ancestral genomes [15, 25] as an ordering of synteny blocks defined through whole genome alignment of the extant genomes [26] can create a more contiguous genome structure. The length of such blocks can be controlled, and is typically defined to be greater than 100 kb. At this resolution, it is common to assume that synteny blocks appear at most once in a descendant species for amniotes [27], and at most once in the ancestral reconstruction. These reconstructions usually have low fragmentation (MGRA [17], for example, produces exactly as many fragments as the maximum number of extant chromosomes). However, micro-rearrangements occurring within each synteny block [4] are hidden, preventing us from obtaining a comprehensive view of the genome evolution at this level.

In this paper, we propose a new heuristic framework, MULTIRES, that integrates information from multiple resolutions to reconstruct the ancestral genome. The method uses reconstructed synteny block orders of an ancestor to infer gene orders while incorporating duplications. MULTIRES uses an approach described in [24] for finding circular chromosomes in the presence of duplications. We develop a novel method for partitioning families of homologous genes using the synteny blocks that they occur in. We show that MULTIRES recovers up to 18 *%* more ancestral adjacencies that are missed by a method which uses the same optimization routine without using synteny blocks (originally implemented for scaffolding ancestral contigs in [28]) on simulated data, and provide a more comprehensive reconstruction of the X-chromosome of the primate-rodent common ancestor.

## Method

We assume that we are given the following pieces of data as input.

1. A resolved (binary) phylogenetic tree on a set of extant species, and a marked ancestral node at which we want to reconstruct the genome. We are also given branch lengths on the tree. In the absence of branch lengths, we may assume that each branch has length 1.
2. A set of ancestral synteny blocks on the extant species. These blocks capture genomic regions across different genomes with high sequence similarity, and can be defined by comparing multiple genomes [15, 26]. It is assumed that all homologous extant synteny blocks evolved from a single ancestral region [27].
3. Extant gene orders, with genes grouped into homologous *gene families* consisting of orthologous and paralogous genes in all species.

Our aim is to reconstruct the gene order at a given ancestor of interest. The challenge here is twofold: given two homologous genes, we need to distinguish where they appear on the ancestral genome, and the gene order needs to be ‘consistent’ with the ancestral synteny block order. In this paper, we define consistency as finding a gene order such that, for each consecutive subsequence *W* of synteny blocks and gaps between the blocks which is inferred to exist in the ancestral genome, there exists a corresponding consecutive subsequence *S* in the gene order such that the genes and adjacencies in *S* are preserved within *W*, according to some parsimony criterion which we specify later. We want to find the largest weight set of gene adjacencies which is (i) consistent, with weights defined by the status of their phylogenetic conservation [15], while ensuring that (ii) the number of copies of each gene in the order is upper bounded by a precomputed ancestral copy number. MULTIRES is presented as a heuristic that aims to achieve both.

**Fig. 1:**
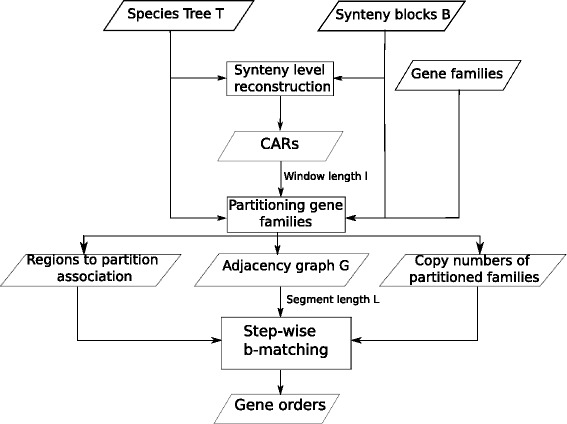
MULTIRES flowchart. A high-level overview of the MULTIRES pipeline

The outline of MULTIRES is presented as a flowchart in Fig. 1. We first infer an ancestral order for the synteny blocks. We use ANGES [29] to find an ancestral reconstruction using the synteny blocks and the species tree as inputs. Note that it is possible to use different methods for this purpose. We used ANGES since, at the time of the experiments, it was one of the few software that could consider non-unique, non-universal synteny blocks in the extant species and produce an ancestor with at most a single copy of each block. Since then, we also have the option of using other methods, such as the new version of MGRA [19], but the results were identical to those of ANGES on the X-chromosome data set and simulations in our experiments. We use the set of *contiguous ancestral regions* (CARs), sequences of synteny blocks, obtained from ANGES as an input.

**Fig. 2:**
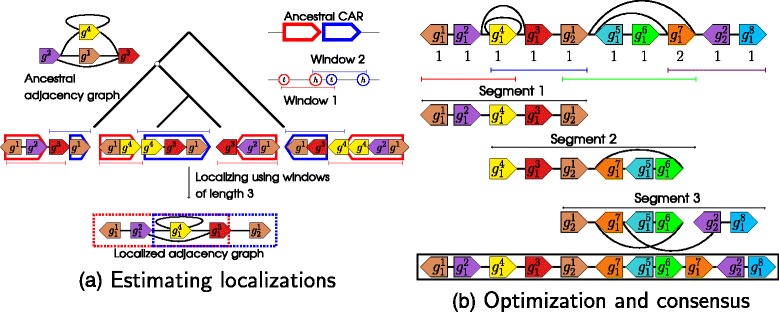
**a** Estimating localizations. Here it shows how MULTIRES defines and infers *localizations*. Coloured solid wedges represent gene families, with wedges of the same colour belonging to the same family. Synteny blocks are indicated by hollow wedges, with colour indicating homology. The orientation of the wedges represent the orientation of the genes/blocks. Inferring adjacencies between gene families parsimoniously results in the *ancestral adjacency graph* shown on the top left, with edges representing adjacencies between gene ends. We also have a set of *contiguous ancestral regions* (CARs) reconstructed at the ancestor, each of which consists of an ordering of the ancestral synteny blocks. On the right of the tree, we display an example of a CAR, and the CAR after all synteny blocks have been doubled into head and tail extremities. We define *windows* of length 3 as consecutive subsequences of 3 extremities on the CAR. The windows, indicated by coloured line segments in the figure, are used to partition the CAR. In the diagram, we observe after partitioning that one copy of the brown gene (*g* ^1^) always occurs in the red window, and one always occurs in the blue window, and never in their intersection. This allows us to partition the brown gene family into two subfamilies, ${g^{1}_{1}}$ and ${g^{1}_{2}}$, called *localizations*, which are restricted to appear only in the relevant blocks, leading to the *localized adjacency graph* at the bottom. **b** Optimization and consensus. Here we show a localized adjacency graph (top) with copy numbers associated to each localization (numbers under the genes). Partitioning the ancestral CARs into segments (black line segments) defines an ordered sequence of induced subgraphs. Using the algorithm given by [24] on each induced subgraph results in a set of adjacencies shown at each layer, with each localization adjacent to at most as many adjacencies as its copy number. For example, the brown localization ${g_{2}^{1}}$ can have at most 1 copy in the gene order, making it adjacent to at most 2 other localizations. The algorithm indicates that these adjacencies are to the red (${g^{3}_{1}}$) and orange (${g^{7}_{1}}$) localizations. Finally, we combine the subgraphs and find a linear gene order by finding the most frequently conserved adjacencies and using the order of the segments. In the example, since the purple localization ${g_{2}^{2}}$ is only conserved in Segment 3, while the cyan localization ${g_{1}^{5}}$ is conserved in Segment 2 as well, we can resolve the gene order around the duplicated orange localization ${g_{1}^{7}}$

The main idea of MULTIRES is summarized in Fig. 1, and along with some accompanying notation, is presented as a schematic view in Fig. 2. We use the mapping of gene families into synteny blocks on the extant species to partition a gene family into one or more subfamilies, called *localizations*, which are expected to be sufficiently far apart in the ancestral reconstruction. We construct an *ancestral adjacency graph* on the set of localizations, using the set of adjacencies between families which are conserved within consecutive subsequences in the CARs. All adjacencies in this graph are given a weight based on their conservation pattern in the species tree, as defined in [15]. We then use the algorithm presented by Maňuch et al. in [24] to find a maximum weight set of adjacencies such that nodes of specified subgraphs can be arranged into a set of circular sequences, with constraints on how many times each node may appear over all sequences. To the best of our knowledge, this is the only known polynomial time algorithm which outputs an optimum weight set of adjacencies with a set of chromosomes with duplications as input. Finally, we combine the results for all subgraphs to obtain a linear gene order for each CAR.

In the interest of brevity, we only present the framework after the ancestral reconstruction at the synteny block level. The inputs we use are the phylogenetic tree, extant gene orders, a set of adjacencies between synteny blocks, ancestral gene copy numbers, and a set of CARs. For details of the process used to obtain the input, see the Additional file 1. We also refer the readers to [16, 30] for reference on how to compute conserved characters and ancestral copy numbers.

### Definitions

We first introduce some common terminology. A *gene*, in the context of this manuscript, is a short contiguous segment of a genome. For a given set of extant species, we assume that we know the exact order of genes in their genomes. A *gene family* is a set of genes, either within a single species or across a set of extant species, which are inferred to have evolved from a single original gene in some ancestral species. The set of gene families forms a partition of the set of genes.

Each gene family can be partitioned into a *head* and a *tail*, following the usual method of *doubling* [12, 31]. The head and the tail of a gene family are referred to as *markers*, and there is a one-to-one relation between a given head/tail marker and the associated gene family. Thus, a chromosome can be thought of as a sequence of markers, not necessarily unique, with length 2*n* (*n* being the number of genes in the chromosome, with a unique pairing of the markers in positions 2*k*−1 and 2*k*, *k*∈{1,…,*n*}). Two successive markers in the sequence that are not paired to each other are said to form an *adjacency*. It is possible for two markers corresponding to extremities of the same gene family to not be paired to each other; these correspond to tandem duplications. Given markers *g*,*h*, an adjacency between them is denoted by {*g*,*h*}. However, for the purposes of exposition, we will represent genes in examples and figures as a single solid element instead of a combination of 2 point markers.

Given a gene family *g*, or equivalently a marker *g*, an *occurrence* of *g* on an extant genome denotes a specific locus at which a gene belonging to this gene family/marker occurs. Each gene family and the corresponding markers are associated to a precomputed ancestral copy number [30]. This number defines an upper limit on the number of copies of the gene in the ancestor.

Synteny blocks can also be doubled, and give rise to two *extremities*. As in the case of markers, a chromosome can also be defined as a sequence of extremities of length 2*n*, with a unique pairing of extremities in positions 2*k*−1 and 2*k*, *k*∈{1,…,*n*}, to form synteny blocks. We use the term *region* to denote a pair of two extremities. Thus, a region represents either a pair of extremities from the same synteny block, or the pair of extremities which frame a gap between two adjacent synteny blocks. We will use the notation [*a*,*b*] to denote a region, where *a*,*b* are block extremities. From now on, we will only work with extremities and regions in order to take into account the orientation of the blocks.

A *contiguous ancestral region* (CAR) *C*=*c*_0_.….*c*_2*k*−1_ of length 2*k* is a sequence of 2*k* extremities *c*_*i*_, such that each region [*c*_2*i*_,*c*_2*i*+1_] is a synteny block, and each region [*c*_2*i*−1_,*c*_2*i*_] is an adjacency between synteny blocks. By extension, it can also be described as a sequence of 2*k*−1 regions. The output of general ancestral reconstruction techniques is a set of CARs, describing reconstructed contiguous genomic segments in the ancestor.

Consider two markers *g* and *h*. We say that an adjacency {*g*,*h*} is *parsimoniously conserved*(or equivalently, *conserved*) in the ancestor if, for two extant species *S*_*i*_ and *S*_*j*_ in the phylogenetic tree, we find that the two markers *g* and *h* are adjacent in both species, and the ancestor under consideration lies on the evolutionary path between these two species in the species tree. The term *conserved* is also used to refer to a region [*a*,*b*] that occurs in two extant species such that the ancestor of interest lies on their evolutionary path in the species tree.

An *ancestral adjacency graph*(or *adjacency graph*) on the set of markers is a graph *G*=(*V*,*E*), where the vertex set is the set of all markers under consideration, and the set of edges is the union of the set of parsimoniously conserved adjacencies between markers on a given species tree and the set of edges between the head and tail markers of the same gene [15]. It is easy to see that a genome can be described as a set of walks on the adjacency graph, with each walk alternating between the conserved adjacencies and the edges between head and tail markers of the same gene [23, 24]. We use a function $\mu : V\to \mathbb {N}$, called the ancestral *copy number* or *multiplicity* function (see [30] or consult the Additional file 1 for details on how to infer the function). This specifies an upper limit on the number of copies of a single marker (and by extension, of a single gene) allowed in set of walks [23, 24]. We also have a positive weight function $w: E\to \mathbb {R}$, which is inferred from the phylogenetic conservation of each conserved edge [15].

### Estimating extant containments

Given a set of extremities, we can find the sequence of genes that are contained within these extremities in every extant species. For a gene family *g* with an occurrence of length *ℓ* in a given extant species, we say that *g* is contained within an extant region [*a*,*b*] in the same species if at least half the length of the occurrence (i.e., *ℓ*/2) lies within the region [*a*,*b*]. Formally, if *g* has head/tail markers at loci *x*<*y* in a given extant species, we say that the gene family *g* is *contained* within a region [*a*,*b*] in the species, if, given that the extremities of the region are consecutive on the extant genome, and located at loci *l*_*a*_<*l*_*b*_ on a given chromosome, one of the following conditions holds.

1. *l*_*a*_≤*x*<*y*≤*l*_*b*_, or
2. *x*<*l*_*a*_<*y* and |*l*_*a*_−*y*|≥*ℓ*/2 or,
3. *x*<*l*_*b*_<*y* and |*l*_*b*_−*x*|≥*ℓ*/2.

Similarly, if the head and tail markers of *g* are located at loci *x*>*y*, then *g* is said to be contained in a region [*a*,*b*] in the extant species if the symmetrical conditions hold. For each extant region, in each species that this region is found in, we thus obtain the sequence of gene family occurrences in this region, if any. Ideally, the gene family sequence within a region would be conserved across all extant species, and the mapping of these gene family sequences to the synteny-level reconstruction should define an ancestral gene order. However, this is rarely the case in real data due to rearrangements, insertions, and deletions at the gene level within the regions. The subsequent sections address how to find an order of the genes such that the gene content within successive sequences of regions is preserved, and the total weight of adjacencies between the genes in a given sequence, inferred phylogenetically, is maximized.

### Finding gene orders in a CAR

We now use the extant gene sequences in regions, conserved marker adjacencies and copy numbers, the species tree, and CARs as the input to find putative ancestral gene family orders.



#### Inferring conserved adjacencies

Given an ancestral synteny order reconstruction in the form of CARs, a consecutive subsequence of regions in this reconstruction should inform us about the gene content in the corresponding region. To formalize this intuition, we define a *window* as follows.

##### **Definition 1**

Let *C*=(*c*_0_,…,*c*_*k*−1_) be a CAR, where each *c*_*i*_ is an extremity. A *window* of length *ℓ* on *C* is a consecutive subsequence *W*=*c*_*i*_…*c*_*i*+*ℓ*−1_, with each [*c*_*j*_,*c*_*j*+1_], *i*≤*j*<*i*+*ℓ*−1, being a region in *C*. The integer *ℓ* is called the *window length*. A region [*c*_*j*_,*c*_*j*+1_] is said to be *spanned* by the window *W* if *c*_*j*_ and *c*_*j*+1_ are adjacent in *W*.

Fig. 2 a shows 2 different windows of length 3 defined on the ancestral CAR, and the constituent regions as located in a set of extant species. We use windows to partition gene families (and by association, markers) into subfamilies which are expected to occur ‘far apart’ in the ancestral genome. Formally, we have the concept of *localizations*.

##### **Definition 2**

Let *g* be a gene family, and let $\mathcal {W}=\left \{W_{i}:0\leq i< k\right \}$ be a subset of all windows of length *ℓ* in all CARs, such that $\forall \ W_{i}\in \mathcal {W}$ there exist regions *r*,*r* ^′^ spanned by *W*_*i*_, which contain *g* in extant species *S*_*a*_ and *S*_*b*_ respectively, such that *r*,*r* ^′^ are not spanned by any other window in $\mathcal {W}$, and the ancestor of interest lies on the evolutionary path between *S*_*a*_ and *S*_*b*_. Note that *r* and *r* ^′^ may be the same region.

A *localization* of *g* (and by extension, of the markers of *g*), is a subfamily *g*_*i*_ of *g* defined such that all adjacencies to *g* from other gene families conserved within the window *W*_*i*_ are adjacent only to *g*_*i*_.

In other words, if an occurrence of *g* in a region [*a*,*b*] in some extant species is always adjacent to a marker *p*, and an occurrence of *g* in a different region which is not in the same window as [*a*,*b*] is adjacent to a distinct marker *q*, then we can partition the gene family of *g* into occurrences adjacent to *p* and occurrences adjacent to *q*.

Algorithm 1 describes how to define localizations and adjacencies between them. As output, we obtain partitions of the gene families (markers) into localizations, which we denote by *V*, a set of parsimoniously conserved adjacencies between localizations, denoted by *E*, and a function $\mu : V\to \mathbb {N}$, which assigns a copy number to each localization using the parsimony algorithm detailed in [30]. The sets *V* and *E* are used to define a *localized adjacency graph* *G*=(*V*,*E*), which differs from the original adjacency graph on the set of markers in that the vertices are now localizations. The algorithm is summarized in Fig. 2 a, which shows the locations of the windows and their constituent regions in the extant species, as well as a description of how the adjacency graph on localizations differs from the adjacency graph on markers.

The algorithm updates the copy number function *μ* so that the total copy number of all localizations of a given gene family is equal to the original estimated copy number of the gene family. This constraint is enforced by the following heuristic: (i) delete localizations which are not involved in any adjacencies, and (ii) decrease the copy number of the localization with the highest copy number iteratively till the condition is satisfied. The algorithm also associates each localization to a set of regions in which they could be contained in the ancestor. This is represented as a map *ψ*:*V*→2^*R*^, where *V* is the set of localizations, and *R* is the set of all regions, such that for any localization *v*_*k*_∈*V* of a marker *v*, *ψ*(*v*_*k*_) is the set of regions in which *v* can be found in some extant species, as observed in Line 7 of the algorithm. Since each region in *ψ*(*v*_*k*_) can only be associated with a single localization of *v*, we can define the “inverse map” *ψ* ^−1^([*a*,*b*]) as the set of localizations associated to the region [*a*,*b*].

#### Optimizing within a segment

Given the adjacency graph *G* on the set of localizations, and a set of associations of these localizations to regions, we can use the linear structure of the CARs to design a local optimization scheme. In order to do this, we again consider consecutive subsequences of *L* extremities, or equivalently *L*−1 regions, on the CARs, and try to find local gene orders in each of these subsequences. These subsequences, which we will call *segments*, are thus similar to windows, except that the user-defined parameter specifying their length *L* is required to be at least as long as the window length *ℓ* used to construct *G*. Therefore, a segment may contain many windows, and by extension many localizations of the same original gene family. Figure 2 b shows how segments are defined.

Once we have a set of segments of the CARs, we find subgraphs in *G* restricted to the set of regions spanned by each segment and find a ‘good’ set of adjacencies in each subgraph as follows.

1. For each segment of length *L* on a CAR, where the corresponding *L*−1 regions are {[*b*_*i*_,*b*_*i*+1_]}_*k*≤*i*<*k*+*L*_, find the induced subgraph *G* ^′^ of *G* on the following set of localizations,
  $$\begin{array}{*{20}l} V'=\bigcup_{k\leq i< k+L}\psi^{-1}\left(\left[b_{i},b_{i+1}\right]\right), \end{array} $$
  i.e., the set of localizations associated with regions in this segment.
2. Let *μ*(*G* ^′^) be the restriction of the copy number function *μ* to the localizations in *V* ^′^. Use (*G* ^′^,*μ*(*G* ^′^)) as the input to Maňuch et al.’s [24] algorithm to find a maximum weight set of adjacencies in *G* ^′^ which will admit a set of linear or circular chromosomes. The algorithm finds a set of adjacencies in which each localization *v*∈*V* ^′^ is adjacent to at most *μ*(*v*) other localizations.
3. Return the set of all adjacencies found within each segment.

For subgraph *G* ^′^, we obtain a maximum-weight set of adjacencies between localizations such that (i) each localization *v* is adjacent to at most *μ*(*v*) other localizations, and (ii) there is a set of linear/circular walks in *G* ^′^ which uses exactly this set of edges. The set of adjacencies will be similar for consecutive segments, with one possibly extending the other. The definitions of the segments and the result on the associated subgraphs after optimization illustrated in Fig. 2 b.

The previous step is the bottleneck in the process, consisting of multiple maximum matching routines, which take roughly *O*(|*E* ^′^|^3/2^) each, where *E* ^′^ is the set of adjacencies between localizations in a subgraph. However, if the window and segment lengths are carefully chosen, this step can be completed under 400*s* for an instance with ∼730 genes in the extant species, compared to over 1000*s* for other parameter combinations on a single Intel Xeon 2.20 GHz processor, while, as shown in the Additional file 1, varying the parameters does not significantly affect the reconstruction quality.

#### Constructing the final ordering

The final step of the method is to find a consensus sequence of markers using adjacencies kept for each segment of each CAR. We merge the adjacencies kept in each segment to create an adjacency graph for a single CAR. In this adjacency graph, the copy numbers of the localizations are inherited from the previous step, but overlapping segments may have conflicting adjacencies.

We assign each adjacency {*x*,*y*} a weight defined by *w* *g* *t*({*x*,*y*})=*P*({*x*,*y*})/*T*({*x*,*y*}), where *P*({*x*,*y*}) is the number of subgraphs in which the adjacency {*x*,*y*} is kept, and *T*({*x*,*y*}) is the number of subgraphs in which both *x* and *y* are associated to some region, not necessarily the same.

We then greedily delete the lowest weight adjacency such that the degree of the adjacent markers exceeds their copy numbers. Repeating this process results in a set of adjacencies between localizations which have at most as many adjacencies as their copy numbers. We use the following method to find the order of localizations.

1. Rank the localizations based on the sequence of regions they are contained in. For example, a localization is contained in a sequence *c*_0_.*c*_1_.*c*_2_ of extremities is ranked higher than one that appears in the sequence *c*_1_.*c*_2_.*c*_3_ on the same CAR.
2. Starting at the highest ranked localization, if it has a unique neighbour, add the neighbour to the expected path.
3. Traverse the graph in the direction of the neighbour of the last localization added to the path.
4. If a localization has more than 1 neighbour, traverse the graph in the direction of the highest ranked neighbour, taking into account the number of copies of that neighbour used. If all copies of the neighbour have been used, move to the next highest ranked neighbour.
5. If there is a tie in the ranking, construct the paths from the tied neighbours separately, and add them to the path sequence in order of the highest ranked ending vertex in the paths.
6. If the traversal returns a cycle, delete the last edge traversed.
7. Return path(s) obtained in the order of their traversal.

The final order is returned as a set of concatenated paths, with the order and orientation of each path expected to be indicative of the relative order of the markers compared to the neighbouring paths, as shown in Fig. 2 b.

## Results

**Fig. 3:**
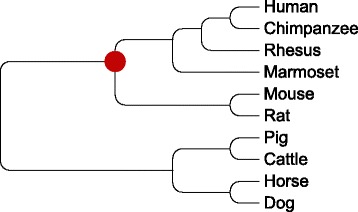
Mammalian species tree. The species tree, with the ancestor of interest marked in red

We used MULTIRES to reconstruct the X-chromosome gene order of the primate-rodent ancestor for both simulated data as well as real data. We used human, chimpanzee, rhesus macaque, marmoset, rat and mouse as ingroups, and dog, cattle, pig and horse as outgroups. The genes and species tree were obtained from Ensembl (the species tree is illustrated in Fig. 3). We used synteny blocks of resolution 100 K on the descendant species, computed using whole genome comparison. No synteny block appeared twice in any species, a common assumption for amniotes [27], but they could be unique to a single descendant.

Unless otherwise mentioned, the method is run with parameters of window length 25 and segment length 65. In the Additional file 1, we show that the parameters in the range chosen do not significantly change the quality of the reconstruction, measured as the number of ancestral adjacencies recovered. However, longer window and segment lengths lead to added complexity, since the induced subgraphs grow larger. Our choice of window and segment lengths is based on minimizing the running time for the chosen parameters.

### Results on simulated data

We created 50 simulated data sets each at 2 different rearrangement, duplication, insertion and deletion rates. The simulation methodology is described in the Additional file 1. The two rearrangement rates were chosen so that the number of breakpoints between ingroup species form lower and upper bounds to those found in the real data. We called the two simulation sets at different rearrangement rates the *low rearrangement simulations* and the *high rearrangement simulations* respectively.

#### Simulation results

We ran MULTIRES for $\binom {8}{2}$ parameter combinations, varying segment lengths and window lengths from 15 to 85 at intervals of 10. We assessed our results by comparing against FPMAG. FPMAG is a method derived from FPSAC [28], a tool for scaffolding ancestral bacterial contigs. While FPSAC and FPMAG are not intended for use on mammalian genomes, they use the maximum matching routine described in [24], and the concept of *repeat spanning intervals* [28] to resolve duplications. To the best of our knowledge, this is the only publicly available software package that computes an ancestral reconstruction in the presence of duplications with only gene families and a phylogenetic tree as input. Since it uses the same optimization routine as MULTIRES, we feel this comparison can be used to gauge how the introduction of synteny blocks can augment the reconstruction process. We also compare MULTIRES against MGRA2 [19], in order to highlight how the presence of duplications can obfuscate ancestral gene order reconstruction.

**Fig. 4:**
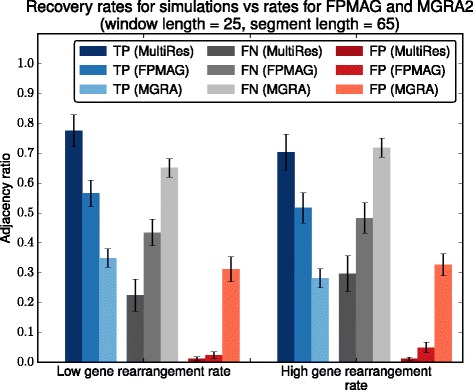
Adjacency recovery comparison. Comparison of adjacency true positive (TP), false positive (FP) and false negative (FN) rates for MULTIRES against FPMAG (cf. [28]) and MGRA2 [19] on both the low rearrangement rate and high rearrangement rate simulations. FPMAG fails to recover a number of ancestral adjacencies, despite using repeat spanning intervals. The results using MGRA2 are provided to contrast how much of a difference the presence of duplications can make in a reconstruction

We used the number of ancestral adjacencies recovered in the reconstruction to measure reconstruction quality. Figure 4 compares the true positive, false positive and false negative rates of reconstructed adjacencies for both simulation sets, using fixed parameter values as recovered by FPMAG, MGRA2 and MULTIRES. MULTIRES yields a significantly longer reconstruction, of average length ∼627 adjacencies for the low rearrangement sets, with ∼78 % true positives, and average length ∼583 for the high rearrangement sets, with ∼70 % true positives. The false positive rate in both cases is well under 10 *%*. In comparison, FPMAG returns a reconstruction with an average of ∼445 and ∼416 adjacencies, with true positive rates of ∼56 % and ∼50 % for the two simulation sets respectively, while MGRA2, which ignores duplications, finds at most ∼35 % true positives and about ∼31 % false positives in reconstructions of average length ∼496 and ∼445 respectively.

Comparing the fragmentation levels of the methods used, we found that FPMAG produces ∼39 CARs (standard deviation = 5.16) in the low rearrangement simulations on average, while in the high rearrangement simulations, it produces ∼45 CARs (s.d. = 6.85). MGRA2 produces only 1 CAR, but does not recover most of the gene content, as seen in Fig. 4. Using both synteny blocks and gene families, MULTIRES finds on average 46 fragments in the low rearrangement simulation set, and ∼59 fragments in the high rearrangement simulation set. However, there is a total order on these fragments, which are linearly ordered on 1 or 2 CARs formed at the synteny block level, for both rearrangement rate sets on average. Therefore, the reconstructed gene order is reconciled with the synteny blocks and their adjacencies which are expected to contain those genes.

#### Larger scale conservation

We also examined the number of recovered common intervals, as defined in [32]. A *common interval* of length 2*k*>2 between two genomes is a set of 2*k* (not necessarily distinct) markers, or equivalently *k* genes, which are found to occur consecutively in both genomes, with the internal order of the genes unspecified.

Here, we do not compare against MGRA2 and FPMAG for the following reasons: the gene content recovered by MGRA2 is comparatively low, which precludes the possibility of recovering a large number of common intervals, and FPMAG has a high fragmentation rate, due to which very few sufficiently long common intervals are recovered. In comparison, MULTIRES has the advantage of having ordered the genes on ancestral CARs, which allows for a better comparison against the simulated ancestral genome.

**Fig. 5:**
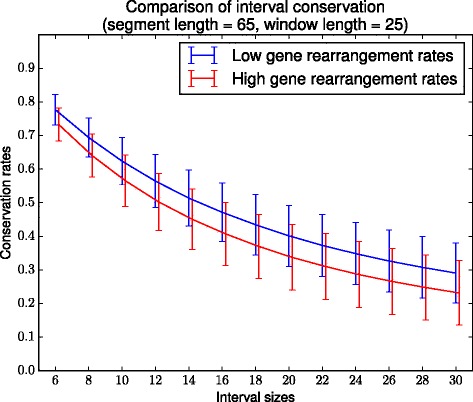
Interval recovery. The ratios of intervals recovered against the size of the intervals, for both simulation sets. Note the steady difference in the ratio of recovered intervals: fewer intervals in the high rearrangement set are recovered. The red plot has been shifted by 0.1 along the x-axis for easier viewing. Longer intervals are lost due to the number of genes which are not recovered in the reconstruction

Comparing the number of recovered intervals at both rearrangement rates for fixed parameter values, we obtained Fig. 5. We first point out how the number of common intervals decreases rapidly with interval length. This is a result of the number of genes in the ancestor that were not found in the reconstruction: since such genes are never found, all intervals containing them are lost. However, the number of short intervals recovered (length 6 to 10) is usually competitive with the number of ancestral adjacencies recovered. Indeed, in the high rearrangement sets, more intervals of length 6 are recovered (≥70 %) than adjacencies, as seen by comparing with Fig. 4. This shows that MULTIRES finds small neighbourhoods of co-localized genes present in the ancestor, even if the exact gene order is hard to recover.

Another reason for the loss of large intervals is our reliance on the synteny-level reconstruction. Any intervals which contain markers from two or more different CARs will be lost. For example, if the synteny level reconstruction produces 2 CARs for a single ancestral chromosome, and of two adjacent genes, each one is found in a region on exactly 1 CAR, then no intervals containing both of them can be recovered.

### Results on real data: ancestral X-chromosome of the primate-rodent common ancestor

For the experiments on the real data, we attempted to reconstruct the X-chromosome of the primate-rodent common ancestor. The X-chromosome was chosen due to the high concentration of gene duplications (∼20 % of the gene families). We used synteny blocks with resolution 100 Kb, and found a synteny-level reconstruction of the ancestor using ANGES consisting of a single CAR of 374 synteny blocks. The set of extant genes consisted of 626 extant gene families occurring in at least 2 extant species, at least one of which is an ingroup species. Gene families with inferred ancestral content greater than 15 were discarded.

As before, we compared the method against MGRA2 and FPMAG. To evaluate the reconstruction, we consider the total number of genes which are recovered in the reconstruction, the number of adjacencies found, and the number of fragments reconstructed. Table 1 summarizes the results on the real data. We found that a large proportion of the total possible gene content was lost. Of 746 possible genes (summing up the ancestral copies of each gene family), we found around 518 genes, with a maximum of 553, and a minimum of 478 depending on the parameters used. Similarly, of 749 conserved ancestral adjacencies, we recovered around 468, with a minimum of 459 and a maximum of 480. This is an adjacency recovery rate of about 62 %. The method found 57 linear fragments on average, ordered on the single CAR to obtain a gene-level representation of the ancestral X-chromosome.

**Table 1:** Comparison of the gene order reconstruction of the primate-rodent ancestral X-chromosome using MGRA2, FPMAG and MULTIRES

	Conserved	MGRA2	FPMAG	MultiRes
Genes	746	132	429	518.12 (19.81)
Adjacencies	749	130	350	468.31 (6.05)
Recovered	-	42	350	468.31 (6.05)
Fragments	N/A	1	79	53.16 (4.76)

The row for total adjacencies indicates the number of adjacencies found in the reconstruction. The third row indicates the number of reconstructed adjacencies which are conserved in 2 or more descendant species. Note that FPMAG and MultiRes only recover conserved adjacencies. MGRA2 can also limit the number of CARs reconstructed and find a single CAR. The results for MultiRes are averaged over all parameter combinations. The low standard deviations demonstrate the robustness of the method to parameter choices

In comparison, MGRA2, which does not take duplications into account, recovered 132 genes, and of the 749 conserved adjacencies, only recovered 42. It also created 88 novel adjacencies which are not phylogenetically supported. However, MGRA2 is able to control the number of fragments created in the process, and finds only 1 CAR.

FPMAG found 429 genes and 350 adjacencies, arranged into 79 CARs. This is an adjacency recovery rate of about 47 %. Both MULTIRES and FPMAG are homology based methods, and do not create any unsupported adjacencies outside those conserved. As in the simulations, the false positives created by these two methods can be attributed solely to convergent evolution scenarios. However, they will also fail to recover ancestral adjacencies which are lost along all branches of the species tree.

## Discussion

The use of low-resolution genomic information in order to improve the accuracy of high-resolution genomic reconstruction is not limited to ancestral reconstruction; for example, using long reads to improve short-read assembly is a well-studied principle [33]. Till recently, though, ancestral reconstruction relied on genomic information at a single resolution. Longer regions were inferred via ancestral conservation [16, 28, 29, 34].

The current method relies on the quality of the synteny-level reconstruction. While this provides the added flexibility of using a given synteny-level reconstruction, if the original synteny-level reconstruction is still highly fragmented, we cannot hope to achieve better results at the gene-level reconstruction. Using the gene-level data to correct the synteny-level reconstruction would be an interesting next step for the current model. A rigorous formalization and analysis of the current model, along with comparison to improved models, could provide useful insights into the robustness of the method and its place in future reconstruction pipelines.

In order to validate the result of the reconstruction, we used both adjacency and interval conservation as metrics. One of the problems we ran into while computing interval conservation was the inability to recover large intervals due to loss of gene content. In this regard, it would be useful to consider the concept of approximate common intervals [35–37]. We aim to analyze interval conservation in this context in future work.

As with other homology based reconstruction methods, MULTIRES cannot detect signals of convergent evolution. In Fig. 4, the false positives found by MULTIRES are all attributable to convergent evolution. In the high rearrangement rate simulations, for example, an average of 213 adjacencies, out of a total of ∼885 conserved in the descendant species were not present in the ancestor. Furthermore, 10 % to 15 % of the false negatives are ancestral adjacencies that were lost during evolution. This problem is exacerbated at higher rearrangement rates.

## Conclusion

The results presented in this manuscript provide a proof of concept on how synteny block information obtained via multiple genome comparison can help ancestral reconstruction at a higher resolution where duplications may be prevalent. The implications of the method are twofold: (i) even with a high level of fragmentation, it is possible to obtain a relative order of the fragments on the synteny-level reconstruction, and (ii) the synteny blocks allow us to disambiguate duplications, which are normally discarded in reconstruction methodologies, thus preventing fragmentation and obtaining a more complete reconstruction. From a methodological point of view, the method described relies on the decomposition of the reconstruction problem into many smaller, overlapping subproblems, which to our knowledge is a novel technique in ancestral reconstruction. The use of the maximum matching routine [24] for these subproblems instead of on the whole graph also allows us to better control the linearity of the result obtained, preventing the reconstruction of large, circular components.

The approach introduced in MULTIRES provides a proof of principle for further development that takes into account information from different resolutions to achieve more comprehensive ancestral genome reconstruction.

## Additional file

Additional file 1 Supplementary Material: Additional background to the methods used, and additional figures showing the variation in MultiRes results with parameters, and runtime. (PDF 303 kb)
