# Supplementary material for: Reconstructing ancestral gene orders with duplications guided by synteny level genome reconstruction
Source: BMC Bioinformatics. 2016 Nov 11;17(Suppl 14):201–12. doi: 10.1186/s12859-016-1262-8 (PMC5123302; doi:10.1186/s12859-016-1262-8)
Supplement: Supplementary file 1 — Supplementary Material: Additional background to the methods used, and additional figures showing the variation in MultiRes results with parameters, and runtime. (PDF 303 kb) [file 12859_2016_1262_MOESM1_ESM.pdf]

Reconstructing ancestral gene orders with duplications guided by synteny level genome reconstruction: Supplementary Material

Ashok Rajaraman and Jian Ma
Computational Biology Department, School of Computer Science
Carnegie Mellon University
Pittsburgh, PA 15213, USA.

S.1 Introduction

This document presents the preliminary steps for processing the input to MultiRes , and explains the main optimization routine used [1]. It also contains figures showing the variation of the performance and runtime of MultiRes with changes in the parameter.

S.2 Methods

S.1 Reconstructing the ancestor using synteny blocks

An initial preprocessing step in the method is to use the phylogenetic tree and the synteny blocks to find a representation of the ancestor by ordering the synteny blocks. We use ANGES [2] as the preferred software to do this, but ideally, any other ancestral reconstruction software, such as inferCARs [3], MGRA [4] or ProCARs [5]. In each case, the output is a set of contiguous ancestral regions (CARs), each of which corresponds to a reconstruction of the ancestral synteny order. For the runs presented in this paper, we use ANGES [2], which works on the concept of *consecutive-ones matrices*. In contrast, MGRA, for example, tries to minimize rearrangement distances between the extant genomes under a certain model.

Our choice of method may affect the final reconstruction, since it uses the synteny-level reconstruction to guide the gene-level scaffolding step. However, in this paper, we consider experiments on the mammalian X-chromosome only. We used both ANGES and MGRA2 [6] to reconstruct the ancestral X-chromosome using blocks of resolution 100K. The main difference is that MGRA2 filters out markers which are not present in all reference extant species, while ANGES does not need to do any such filtering. As a result, we used the reconstructions provided by ANGES for our experiments.

It is important to note that almost none of these methods is suitable for reconstructing synteny/gene orders in the presence of duplications. In the data sets we work on, the mammalian X-chromosomes, almost 20% of the gene families have duplicated occurrences in multiple extant species, with copy numbers as high as 10.

S.2 Finding conserved ancestral adjacencies

In order to find conserved ancestral adjacencies and weight them, we use the following rule: if an adjacency between two markers, or between two block extremities, is present in two extant species, and the unique path between them on the phylogenetic tree contains the ancestor of interest, then we infer the adjacency as conserved in the ancestor. This is a very conservative criterion, and we expect to recover many false positives. However, since we are optimizing over the set of adjacencies later, this is not a particularly troubling issue.

A conserved adjacency between two markers is assigned a weight based on its conservation pattern in the extant species. In order to compute this weight, we use the weighting function available in ANGES [2], which itself is based on the method developed in [3].

S.3 Inferring ancestral copy numbers

Ancestral copy numbers are computed using Wagner parsimony [7] with equal costs for gain and loss of gene copies. More specifically, we consider the number of genes in a single gene family for each extant species, and use a dynamic programming approach to compute the minimum cost ancestral scenario at the root. Then, by backtracking, we find the best cost scenario at the ancestor of interest. The markers inherit the copy numbers of the respective gene families.

It is important to note that we merely find an upper limit to the copy number in the ancestor. For example, if a gene family, and by extension, a marker, is said to have copy number k in the ancestor, we may use fewer than k copies of the marker in the reconstruction. However, it has not been possible to implement a lower limit to the copy number in the method yet. This is left open as a theoretical challenge.

S.4 The optimization scheme

A key part of our method is the use of an algorithm mentioned in [1]. Since this allows the incorporation of duplicated genes, we have chosen it as the preferred optimization algorithm in the new method. In this algorithm, given a graph $G = (V, E)$, where V is a set of genes or gene extremities, and E is the set of adjacencies between them, and $\mu \colon V \to \mathbb{N}$, where μ provides an upper bound on the number of copies of each gene, the output is a subset $E' \subseteq E$ such that

1. for each gene (extremity) $v \in V$, there exist at most $2\mu(v)$(for extremities, $\mu(v)$) edges in E' with v as an end,

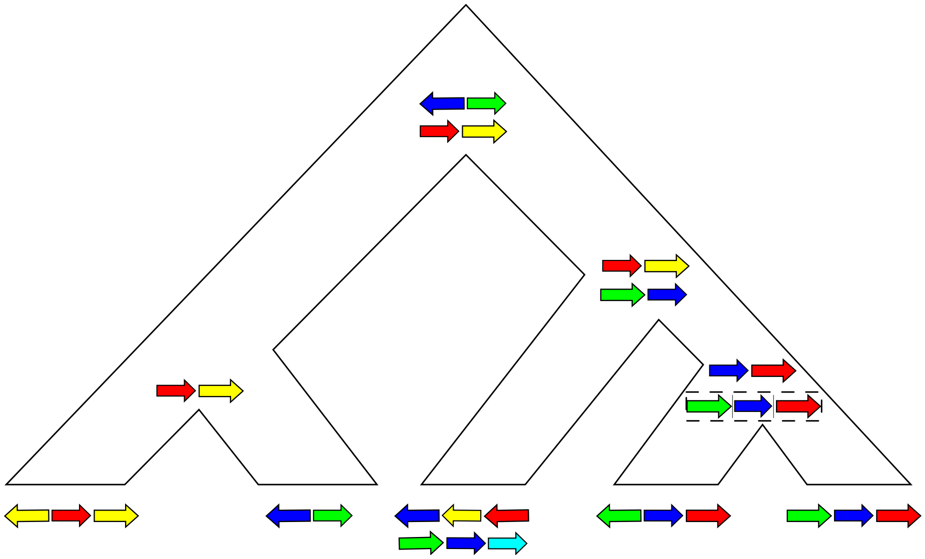

Figure S.1: An example of how adjacencies between genes and synteny blocks are inferred using phylogenetic information. In the figure, the gene orders, with genes represented by coloured arrows, in the extant species are given at the leaves, and adjacencies which are inferred are provided at the relevant ancestral nodes.

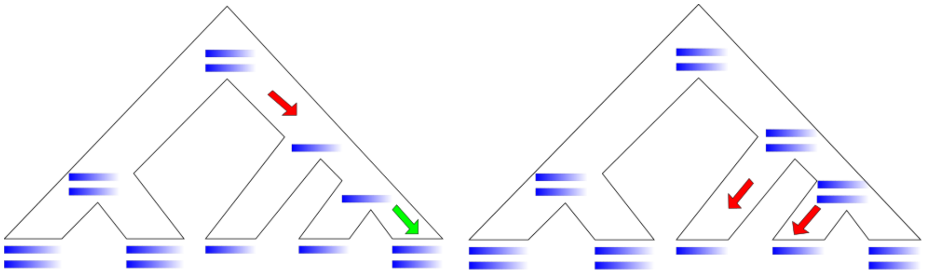

Figure S.2: A schematic showing how copy numbers might be inferred. This is a highly simplified parsimony scheme, in which the number of copies of a gene is provided at the leaf nodes. Losses are depicted by red arrows, and gains by green arrows. We compute a set of annotations for the internal nodes such that the number of gains and losses is minimized. In this example, there are two such scenarios, which are show side by side.

2. there exists a set of linear or circular walks on G which involves only the edges in E', and no v is visited more than $2\mu(v)$(for extremities, $\mu(v)$) times, and

3. there is no subset $E'' \subseteq E$ with these properties which has larger size (or weight, if the edges are weighted).

The algorithm used for this is relatively simple to describe. Let $G = (V, E)$ be the original adjacency graph, with gene families (not extremities) for vertices, and let $\mu\colon V \to \mathbb{N}$ be the copy number function. We first construct a new graph $G' = (V', E')$ as follows.

1. For each gene family $v \in V$, add $2\mu(v)$ copies of v, named $v_0, \ldots, v_{2\mu(v)-1}$ in V'.

2. For each adjacency $e = \{u, v\} \in E$, add 2 vertices e_u and e_v in V'.

3. For each adjacency $e = \{u, v\} \in E$, add the following edges to E': $\{e_u, e_v\}$, and from each v_i (respectively u_i) to e_v (resp. e_u) for $i \in \{0, \ldots, 2\mu(v) - 1\}$ (resp. $i \in \{0, \ldots, 2\mu(u) - 1\}$). Assign them the same weight at $\{u, v\}$, if any, and 1 otherwise.

On this graph, we run a maximum-matching routine, which outputs a set of edges in the matching. Finally, we keep those adjacencies $e = \{u, v\} \in E$ for which we find 2 edges $\{u_i, e_u\}, \{v_i, e_v\} \in E'$ in the matching. This is guaranteed to be a maximum matching, and since we have at most $2\mu(v)$ copies of every gene family v, we are also guaranteed that each gene family has at most $2\mu(v)$ neighbours.

S.3 Supplementary results

S.1 Simulation methodology

We first created a unichromosomal ancestral genome at the root of the tree at both the synteny block and gene resolutions, using the length and copy number distributions of the blocks, genes and gaps in the human X-chromosome. We used an arbitrary cut-off of a maximum copy number of 15 to generate the distributions. Approximately 85% of the gene families found in humans having copy number 1, about 9% of them having copy number 2, and the rest distributed between copy number 3 to 15. About 93% of the duplicated genes have copy number between 2 and 5, so a cut-off above 5 does not affect the distribution significantly. Using these distributions, we evolved the genome along the branches of the phylogenetic tree as follows.

1. At the synteny block level, we performed 3(2) inversions along a pre-ancestral (post-ancestral) branch, with a random breakpoint reuse rate between $[0.05, 0.08)$.

2. For each inversion, we simulated k inversions within the inverted region, where k takes on values from $\{1, 3\}$ for two different simulation sets. If $k = 1$, we label the instance as a *low rearrangement instance*, and otherwise as a *high rearrangement instance.*

3. Over each branch, we duplicated ≤ 8 genes with probability 0.9, and deleted ≤ 2 genes with probability 0.85.

For each rearrangement rate, we created 50 data sets. The parameters for the low rearrangement/high rearrangement simulation sets were chosen so that the number of pairwise breakpoints in the phylogenetic tree between two ingroup species, where breakpoints are defined as adjacencies that are present in exactly one of the two species, is roughly within one standard deviation from the number of breakpoints between the species observed in the real data. The rates of duplications and loss were chosen so that the distribution of copy numbers in the extant human genome created in the simulations is similar to the real data. The average length of the ancestral genome after filtering for copy numbers and by conservation, in terms of the number of adjacencies between genes, was 735 and 756 for the low rearrangement and high rearrangement simulation sets respectively.

S.2 Effect of variation in window length and segment length on the reconstruction

Figure S.4 shows the variation of the true positive, false negative and false positive rates of the number of ancestral adjacencies found in a reconstruction by MultiRes for the two simulation sets when the window length and segment length parameters are varied from 15 to 85 at intervals of 10 blocks each. In total, this leads to $\binom{8}{2}$ possible parameter combinations, since the segment length has to be at least as large as the window length. For both rearrangement sets, we do not notice a significant variation in any of the rates. For larger parameter values, the computation over all simulations took too long, and were excluded from the analysis.

S.3 Runtime vs parameters

Figure S.5 shows how the runtime (in seconds) varies with each of the parameters, keeping the other constant, for a single simlation case. In each case, there is a marked and very rapid upward trend with the parameters. Combined with Figure S.4, this strengthens the case we make in the main paper, which shows the results at fixed parameter values.

References

[1] Maňuch, J., *et al.*: Linearization of ancestral multichromosomal genomes. BMC Bioinformatics **13**(19), 1–11 (2012). doi:10.1186/1471-2105-13-S19-S11

[2] Jones, B.R., *et al.*: ANGES: reconstructing ANcestral GEnomeS maps. Bioinformatics **28**(18), 2388–2390 (2012). doi:10.1093/bioinformatics/bts457

[3] Ma, J., *et al.*: Reconstructing contiguous regions of an ancestral genome. Genome Research **16**(12), 1557–1565 (2006). doi:10.1101/gr.5383506

[4] Alekseyev, M., Pevzner, P.A.: Breakpoint graphs and ancestral genome reconstructions. Genome Research (2009). doi:10.1101/gr.082784.108

[5] Perrin, A., *et al.*: ProCARs: Progressive reconstruction of ancestral gene orders. BMC Genomics **16**(Suppl 5), 6 (2015). doi:10.1186/1471-2164-16-S5-S6

[6] Avdeyev, P., *et al.*: Reconstruction of ancestral genomes in presence of gene gain and loss. Journal of Computational Biology **23**(3), 150–164 (2016). doi:10.1089/cmb.2015.0160

[7] Csűrös, M.: Ancestral reconstruction by asymmetric wagner parsimony over continuous characters and squared parsimony over distributions. In: Nelson, C.E., Vialette, S. (eds.) Comparative Genomics: International Workshop, RECOMB-CG 2008, Paris, France, October 13-15, 2008. Proceedings, pp. 72–86. Springer, Berlin, Heidelberg (2008). doi:10.1007/978-3-540-87989-3_6. http://dx.doi.org/10.1007/978-3-540-87989-3_6

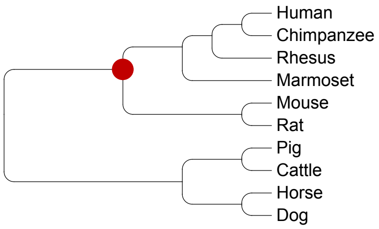

Species	Breakpoints		
	Real data	Low rearrangements	High rearrangements
Mouse	487	456 (148.81)	713 (105.18)
Rat	628	453 (129.14)	696 (105.76)
Cow	615	736 (109.07)	845 (93.52)
Dog	487	724 (98.52)	856 (100.11)
Horse	550	723 (113.34)	845 (95.73)
Pig	582	602 (70.61)	584 (84.68)

Figure S.3 & Table S.1: The figure shows the species tree used, with the ancestor of interest marked with a red dot. The table shows a comparison of the number of breakpoints between humans, and the rodent and outgroup species in the real data and the simulated data, for both sets of rearrangement rates, generated using an ancestor with at most 15 copies per gene. The first column shows the species against which the human X-chromosome, real and simulated, are compared. The parentheses show empirical standard deviations. In order to take into account the large deviation, we provide results for both the low rearrangement and the high rearrangement sets.

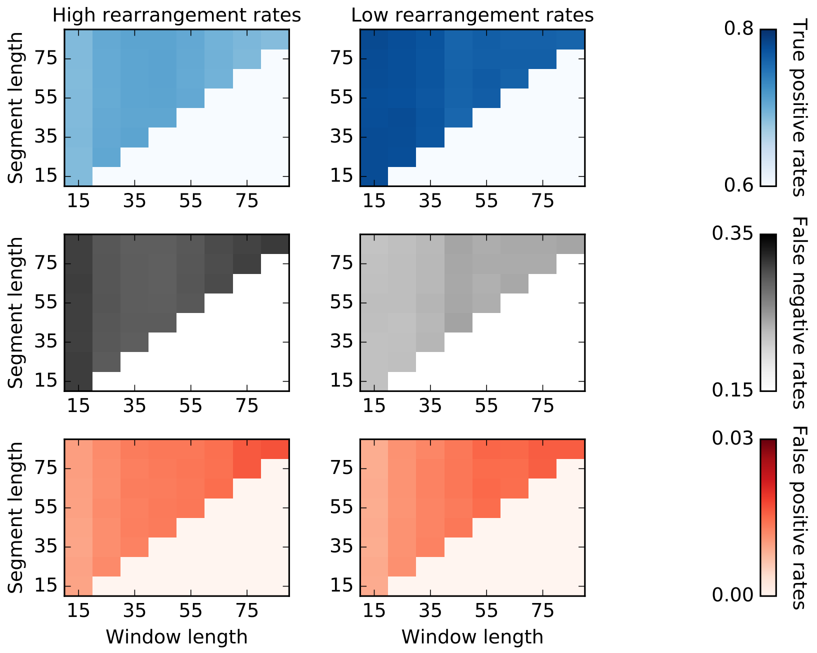

Figure S.4: Comparison of the change in the adjacency conservation with varying window length, on the x-axis, and segment length, on the y-axis. The parameters are varied from 15 to 85 blocks, keeping the segment length greater than or equal to the window length. The number of genes per instance is around 750, and the number of synteny blocks, with resolution 100Kb, is around 380. The three rows correspond to the rate of true positives, false negatives and false positives respectively. The figures in the first and second columns show the effects on the two different simulation sets, simulating high and low rearrangement rates, respectively. In both cases, we note that the difference in the rate of true positives, false negatives and false positives only varies by at most 0.05 points. We also find a stronger banding structure when varying the window length, though there is no clear pattern.

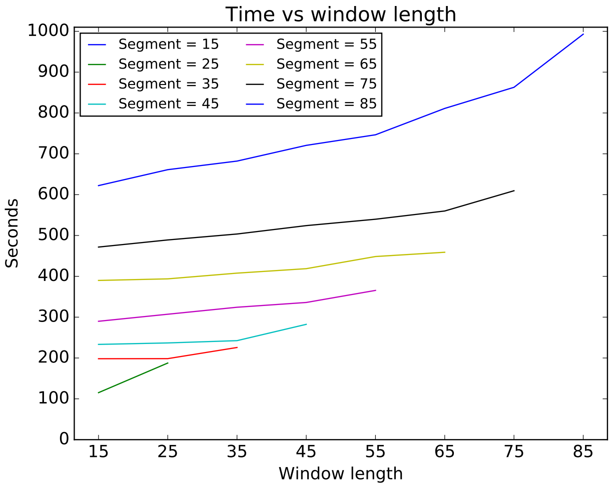

(a) Runtime, in seconds, for a high rearrangement rate example vs window length

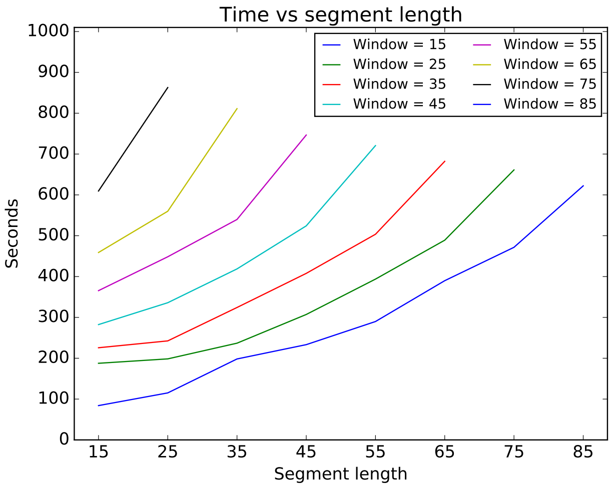

(b) Runtime, in seconds, for a high rearrangement rate example vs segment length

Figure S.5: Variation of runtime with parameters on an input with ~730 genes in the extant species, 396 synteny blocks of resolution 100K, and ~15% of the gene families duplicated.
